# Extracting predictors for lung adenocarcinoma based on Granger causality test and stepwise character selection

**DOI:** 10.1186/s12859-019-2739-z

**Published:** 2019-05-01

**Authors:** Xuemeng Fan, Yaolai Wang, Xu-Qing Tang

**Affiliations:** 1 School of Science, Jiangnan University, Wuxi, 214122 China; 2 Wuxi Engineering Research Center for Biocomputing, Wuxi, 214122 China

**Keywords:** Predictor extraction, Lung adenocarcinoma, Granger causality test, Stepwise character selection

## Abstract

**Background:**

Lung adenocarcinoma is the most common type of lung cancer, with high mortality worldwide. Its occurrence and development were thoroughly studied by high-throughput expression microarray, which produced abundant data on gene expression, DNA methylation, and miRNA quantification. However, the hub genes, which can be served as bio-markers for discriminating cancer and healthy individuals, are not well screened.

**Result:**

Here we present a new method for extracting gene predictors, aiming to obtain the least predictors without losing the efficiency. We firstly analyzed three different expression microarrays and constructed multi-interaction network, since the individual expression dataset is not enough for describing biological behaviors dynamically and systematically. Then, we transformed the undirected interaction network to directed network by employing Granger causality test, followed by the predictors screened with the use of the stepwise character selection algorithm. Six predictors, including *TOP2A, GRK5, SIRT7, MCM7, EGFR*, and *COL1A2*, were ultimately identified. All the predictors are the cancer-related, and the number is very small fascinating diagnosis. Finally, the validation of this approach was verified by robustness analyses applied to six independent datasets; the precision is up to 95.3% ∼ 100%.

**Conclusion:**

Although there are complicated differences between cancer and normal cells in gene functions, cancer cells could be differentiated in case that a group of special genes expresses abnormally. Here we presented a new, robust, and effective method for extracting gene predictors. We identified as low as 6 genes which can be taken as predictors for diagnosing lung adenocarcinoma.

## Background

Lung adenocarcinoma is the major cause of cancer-related deaths worldwide [1–4]. Its occurrence and development follow the changes in complex interactions among genes and their productions [5, 6]. This complexity, presumably, is the main obstacle hindering scientific research and clinical diagnose. The high-throughput technologies provided abundant data on the biological processes [7, 8]. From those data, some key genes were inferred as predictors for classifying tumors and normal samples, substantially fascinating research and diagnose [9].

Most datasets by high-throughput technologies have two shortages in uncovering or describing cellular processes. Firstly, most expression datasets supplied by database such as the Cancer Genome Atlas database (TCGA) [10] do not relate how the functions of genes changes over time, likely with some key information lost. This can be somehow compensated by the time series analysis [11, 12]. Secondly, the expression datasets do not reveal the interactions among genes and their products. This can also be compensated by integrating multiple interaction information at a systematic level such as network analysis [13, 14]. Such systematic integration concentrates more on the molecular interactions rather than the statistical expression differences between cancers and normal samples. So far, network analyses have been widely used in describing bio-molecular interactions, where nodes with higher degree are believed to take more important roles [15].

In the network, gene interactions are complex. For two interacted genes, if one’s expression promotes or represses the other’s expression, it would be termed as “intrinsic causal interaction”. For example, Huang et al. [16] found that *EGFR* mutation enhances expression of *CDH5* in lung cancer cells. That is, gene *EGFR* is the “cause” in their relationship; in other words, *EGFR* is an “independent gene” relates to *CDH5*, while the *CDH5* is a “dependent gene” of *EGFR*. Such causal relation can be obtained by statistical method such as Granger causality test, according to the time series expression datasets. Causality relationship in statistics was initially applied in econometrics, and it is now widely used in determining the regulation directions in biological researches [17, 18]. The directed network obtained by Granger causality test can be further simplified via removing those “dependent genes” while reserving the globally “independent genes”. This represents a way for extracting fewer genes that play dominant roles.

For one disease, genes that express differently compared with the healthy samples are called diff-genes. Of the diff-genes, those that play more significant roles than the others are termed as feature genes. The predictors, which are a subset of the feature genes, are taken as bio-markers for identifying patients. Two important criteria are used to conclude whether a predictor set is proper for fast clinical diagnoses. One is higher prediction precision, and the other is fewer predictor members. Previous studies have made significant contributions for predictor extraction. Cava C et al. [19] conducted a pan cancer analysis for 16 cancer types and found that a few genes could act as predictors to identify tumors. Liu et al. [20] identified 15 hub genes by the weighted co-expression analysis, and validated that the 15 hub genes could discriminate lung cancer vs normal samples. Dai et al. [21] identified 119 mRNA diff-genes utilizing fuzzy granular space theory, with F-value = 0. 7029 and Rand-index *p*= 0.7272. Li el al. [22] identified mRNA feature genes for differentiating the subtypes of breast cancer based on decision tree algorithm. However, further effort should be made to screen fewer predictors from the obtained feature genes for differentiating cancer and healthy samples.

In this paper, we aimed to screen lung adenocarcinoma predictors, with the use of a novel approach. The approach includes three steps as follows. In the first step, differential expression analysis (DEA) was employed to analysis the gene, DNA methylation and miRNA expression microarrays to find diff-genes. Diff-gene interaction network was then constructed, where genes with higher degree would be retained as feature genes. Secondly, using Granger causality test, the undirected feature gene interaction network was transformed to directed network. A stepwise character selection based on Random Forests (RF) model [23] was further proposed to identify predictors from feature gens. In the last step, we tested the prediction capacity of the predictors, by applying to six independent datasets; the results presented excellent accuracy.

## Methods

### Datasets

The gene, DNA methylation and miRNA quantification data were downloaded from the Illumina HiSeq platform on lung adenocarcinoma (TCGA ID: LUAD) in TCGA [10]. The gene data includes 539 tumor and 59 normal samples, the methylation data includes 448 tumor and 45 normal samples, and the miRNA data includes 473 tumor and 32 normal samples. Six gene expression profiles (GSE10072 [24], GSE83213 [25], GSE2088 [26], GSE32863 [27], GSE43458 [28], GSE27262 [29, 30]), which would be used in validation test, were downloaded from the Gene Expression Omnibus (GEO) [31]. More detailed information of the datasets is shown in Table 1. The dynamic gene expression dataset (GSE79210 [32]) was downloaded from the GEO database, which records the gene expression level at 26 time points (0h, 0.5h, 1h, 2h, 3h, …, 22h, 23h, 24h).

**Table 1:** Basic characteristics of 7 datasets

Characteristics		Analysis	Validation					
		TCGA	GSE10072	GSE83213	GSE2088	GSE32863	GSE43458	GSE43458
	Platform	Illumina	Affymetrix	Illumina	Illumina	Illumina	Affymetrix	Affymetrix
	Cancer/Normal (Total)	539/59 (598)	50/57 (107)	11/46 (57)	57/30 (87)	58/58 (116)	80/30 (110)	25/25 (50)
	Male (%)	107 (18)	69 (64)	28 (50)	Unknown	26 (22)	Unknown	Unknown
Race	Asian	8	0	0	0	44	0	0
	Black	59	0	0	0	0	0	0
	White	446	0	0	0	72	0	0
	Unreported	66	107	57	87	0	110	50
	Mean age	65	56	Unknown	Unknown	68	Unknown	58
	Never-smoker (%)	34 (6)	36 (34)	Unknown	Unknown	Unknown	70 (64)	Unknown

### Feature genes screening

In this study, the differentially expressed genes (DEGs), differentially methylated DNA (Dmets) and differentially expressed miRNAs (DEmiRNAs) were obtained by DEA for each kind of microarrays. The process included a t-test and a log2 fold change for tumors and healthy samples. To reduce the type I error, the *p*-value of t-test must be adjusted. In this paper, TCGAbiolinks [33] package was applied to download data from TCGA and do DEA (cutoff: logFC.cut =1 and FDR.cut = 0.01 for gene and miRNA data; p.cut = 0.01and diffmean.cut = 0.35 for methylation data). To gather diff-genes, we searched the genes with Dmets (i.e., differentially methylated genes; abbr., Dmet-genes), according to the annotation information. Meanwhile, we also searched the target genes of DEmiRNAs (abbr., DEmiRNA-target-genes). Considering that not all genes regulated by DmiRNAs are diff-genes, a miRNA-target network was constructed and the genes with higher degree were identified as DEmiRNA-target-genes. Genes included in any one of the three gene sets, namely the DEGs, Dmet-genes, and DEmiRNA-target-genes, were identified as diff-genes. Supplementarily, the miRNA target information from GeneMANIA [34] database was obtained by SpidermiRNA [35] package, and the network topological analysis was achieved by Cytoscape [36] software.

An undirected multi-interaction network of diff-genes was then constructed to obtain feature genes. Such network integrated three kinds of gene-gene interactions: co-location interaction, physical interaction, shared protein domain interaction. Genes with higher degree were identified as feature genes. According to GeneMAINA (http://pages.genemania.org), the definitions of the three kinds of interactions are as follows. (i) Co-localization interaction: two genes are linked if they are both expressed in the same tissue or if their gene products are both identified in the same cellular location. (ii) Physical interaction: two gene products are linked if they are found to interact in a protein-protein interaction study. (ii) Shared protein domains: two gene products are linked if they have the same protein domain. All the above interaction information is available by SpidermiRNA package.

### Gene predictors extraction

We found the feature genes, whose number was 148, are sufficient for differentiating tumors. However, the number is too large in clinical diagnose. Hence, we developed a two-step method to select some genes from the feature genes, which would be served as predictors without losing accuracy.

#### Setp1: Granger causality test

Granger causality test is a statistical test with the hypothesis that the past of one’s performance is helpful in predicting the future of the other’s performance. More precisely, for variables A and B, if A is the granger causality of B, two conditions must be met: (i) A is helpful in predicting B; (ii) B is not useful apparently in predicting A.

**Fig. 1:**
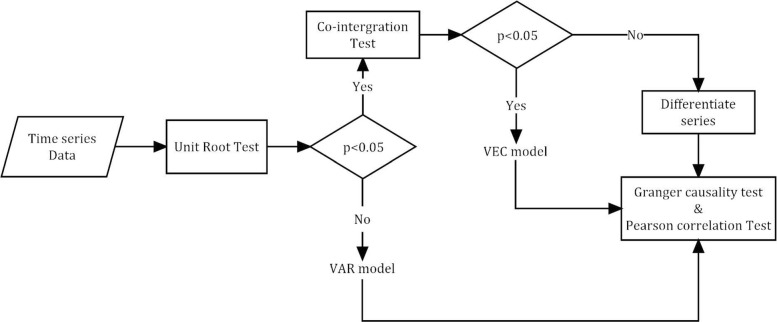
Flow chart of Granger Causality Test. The Pearson correlation test adapts *p*-value < 0.01 as threshold, and the other three: the unit root test, co-integration test and Granger causality test adapt *p*-value < 0.05 as threshold

Although Granger causality test is widely used, it is not enough to assert causal relation in reality, and the verification of such relation requires mass of validated biological information. Inspired by the interaction network, we made a restriction that only two interacted genes would be used as input of the Granger causality test rather than all couples of the feature genes. In addition, Pearson correlation test was also performed to ensure the correlation between genes in expression. Steps of Granger causality test are presented in Fig. 1,

We began with distinguishing two genes with causal interaction by defining a “dependent gene” and an “independent gene”. For two genes *G*_1_ and *G*_2_, *G*_1_ is the “independent gene” of *G*_2_ or *G*_2_ is the “dependent gene” of *G*_1_, only if the two genes satisfy three criteria: (i) *G*_1_ and *G*_2_ are interacted feature genes; (ii) *G*_1_ is the granger cause of *G*_2_; (iii) *G*_1_ and *G*_2_ show significant Pearson’s correlation in expression. In a view of simplification, a couple of independent-dependent genes can be taken as its independent gene.

**Fig. 2:**
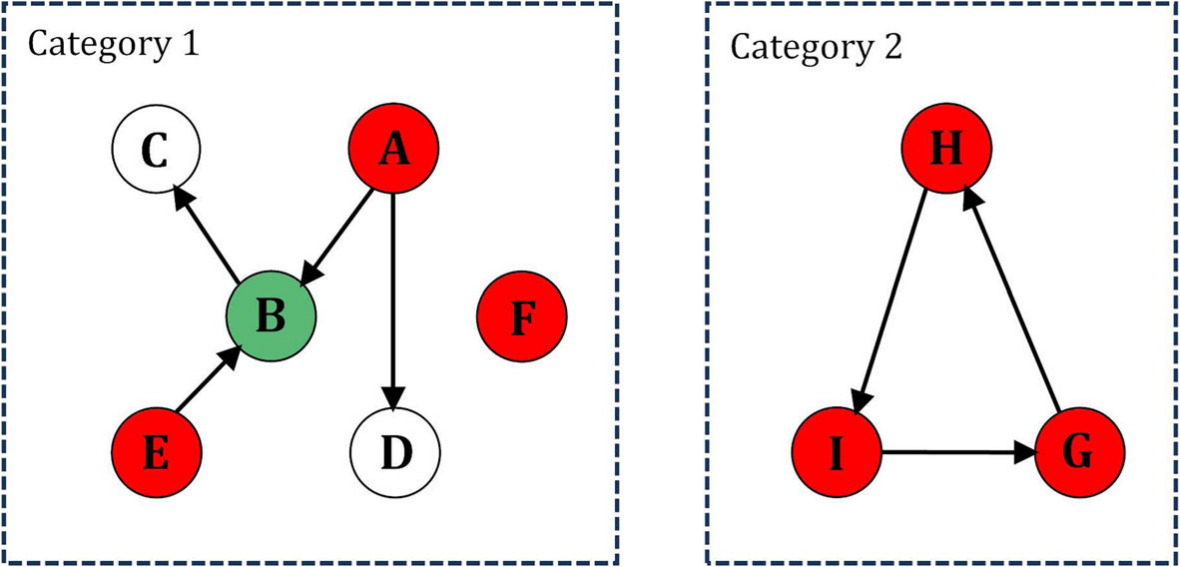
Illustration of how to select the globally independent gens. *A* is the independent gene of *B* and *D*; *B* is the independent gene of *C*, *B* is also the dependent gene of *E*; *F* is a single node gene; *H*, *I* and *G* are in a feedback sub-network. In category 1, we identified *A*, *E* and *F* with indegree = 0 as the globally independent genes (marked in red). In category 2, *H*, *I* and *G* are all identified as the globally independent genes (marked in red). The nodes in green indicate genes that are both dependent genes and independent genes of some other genes. The blank nodes indicate genes are only dependent genes of some other genes

There were 207 causal gene couples in the feature gene network. The network could be simplified via screening the globally independent genes which paly dominant roles in the whole directed causal network. The screening was executed with the following scheme. We observed that there were two categories of topologies in the network as shown in Fig. 2. In the first category, genes either belong to feedforward sub-networks or are isolated genes that do not interact with the others. Both the isolated genes and the source genes (whose in degree is 0) in feedforward sub-networks are taken as the globally independent genes. In the second category, the genes are in feedback sub-networks. All such genes were reserved as globally independent genes.

#### Step2: Stepwise predictor selection based on Random Forest

**Fig. 3:**
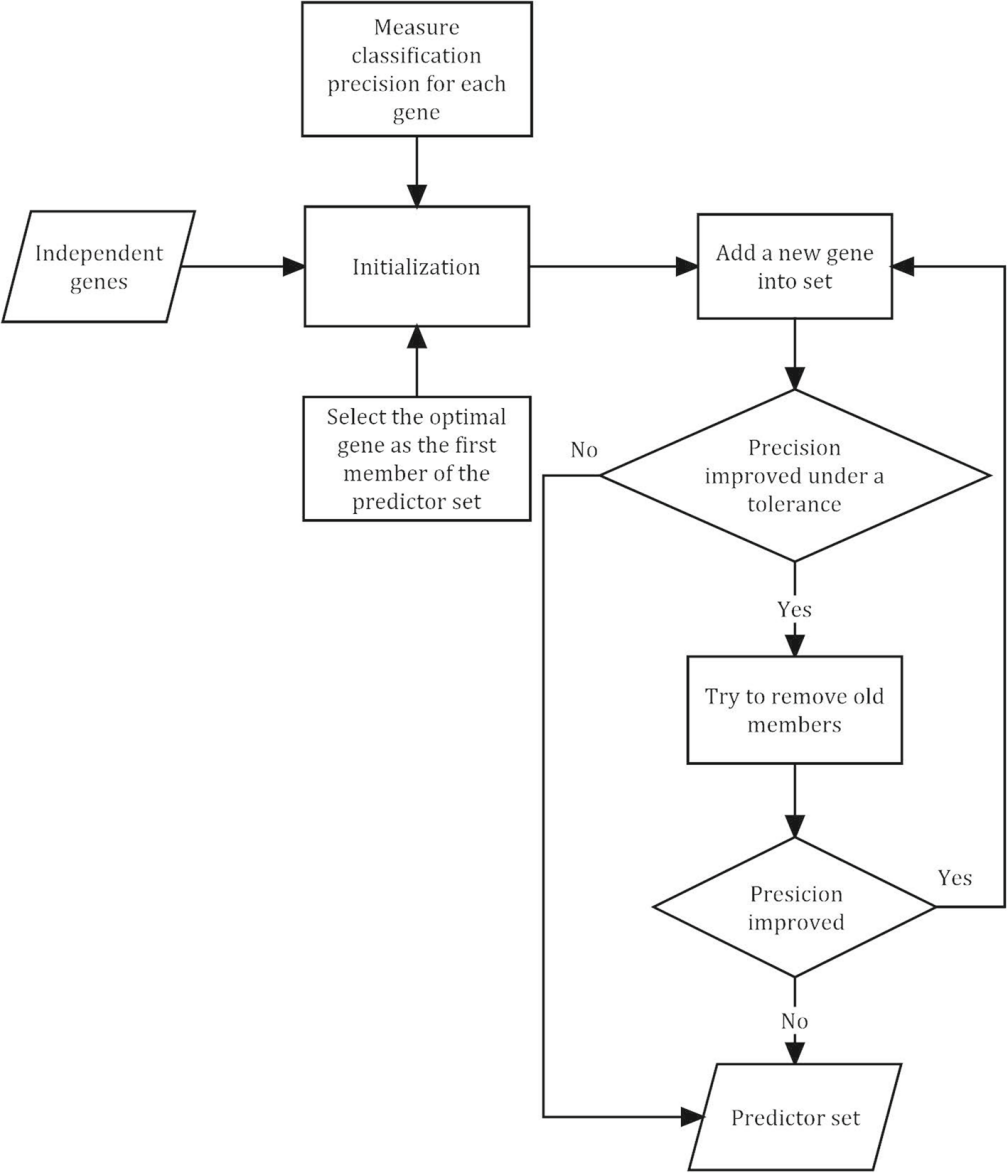
Flow chart of stepwise character selection based on RF

Despite of the well performance in classification of the globally independent genes, there were still 63 genes which is too many for diagnose. We thus proposed a stepwise character selection algorithm, aiming to exact predictors from the globally independent genes without reducing the classification precision. In the first step, we performed a initialization by evaluating the performance of classification for each candidate gene and ranked the candidate genes by precision. In the second step, a new candidate gene was added into the current predictor set. If the accuracy of the predictor set was improved than the last step, then we tried abandoning several old predictors whose removal led to higher accuracy. If the accuracy was still improved after the removal, then we turned to the second step. Such procedure was repeated until there were no new candidate genes or the precision was reduced. The classification was completed by Random Forest (RF) classification model, which is a famous ensemble learning algorithm. Achievement of RF relied on randomForest package and parameters were accepted as ntree (number of tree) = 500 and mtry =sqrt (p), p is the number of variables. Workflow illustrated above is shown in Fig. 3 and summarized in Table 2.

**Table 2:** Process of stepwise character selection based on RF

**Algorithm:** Stepwise Character Selection
**Input:** Ranked independent gene list *G*, number of candidate genes *n*, gene expression microarray
*D*∈*R*^(*n*∗*d*)^,threshold *ε*.
**Output:** Predictor set *P*, predict accuracy *ACC*,*P*_*ACC*.
**Step1:** Initialization: *P*=*∅*, candidate gene set *C*=*G*.
**Step 1.1:**Calculate the accuracy *ACC*_*i*_, *SN*_*i*_,*SP*_*i*_,*MCC*_*i*_ by Eqs. 1, 2, 3, 4 of each *c*_*i*_∈*C*
which acts as a single predictor in RF with 5-fold cross validation, respectively.
**Step 1.2:** *P*←*p*,where $p \leftarrow \mathop {argmax}\limits _{c_{i} \in C}ACC$, $P\_ACC \leftarrow \mathop {max}\limits _{i\in {1 \cdots n}}ACC,C \leftarrow C/p$.
**Step2:** Character selection.
While *P*_*ACC*−*ACC*_*max*>−*ε* and *C*≠*∅*, do
**Step2.1:** *ACC*_*max*←*P*_*ACC*.
**Step2.2:** Add members into *P*.
1.$P_{add\_i} \leftarrow P \cup \{ c_{i} \in C\}$ calculate ${ACC}_{add\_i}$ using $P_{add\_i}$,as predictors in RF with 5-folf cross validation, *i*=1,⋯,*n*.
2.$P \leftarrow P_{add\_I_{add}}$,where $I_{add} \leftarrow \mathop {argmax}\limits _{i=1,\cdots,n}({ACC}_{add}),P\_ACC \leftarrow max(ACC),C \leftarrow C/P$.
**Step2.3:** Try remove members form *P*.
If *P*_*ACC*−*ACC*_*max*>−*ε*,do
1.Define *n*_*remove*_ as the length of *P*.
2.$P_{remove\_i} \leftarrow P/p_{i} \in P$,calculate ${ACC}_{remove\_i}$ using $P_{remove\_i}$as predictors in Random Forest with 5-fold cross validation, *i*=1,⋯,*n*_*remove*_.
3.$P\leftarrow \{p_{i}|{ACC}_{remove\_i}>P\_ACC,i=1,\cdots,n_{remove}\}$.
$C \leftarrow \{C \cup \{p_{i}|{ACC}_{remove\_i} \leq P\_ACC,i=1,\cdots,n_{remove}\}$
End if.
**Step2.4:** Calculate accuracy.
Calculate *P*_*ACC* using *P* as predictors in RF with 5-fold cross validation.
End while.

Four accuracy indexes were calculated to measure the performance of a predictor set in the stepwise character selection algorithm:

1 $$\begin{array}{@{}rcl@{}} ACC &=& \frac{TP+TN}{TP+FP+FN+TN} \end{array} $$

2 $$\begin{array}{@{}rcl@{}} SN &=& \frac{TP}{TP+FN} \end{array} $$

3 $$\begin{array}{@{}rcl@{}} SP &=& \frac{TN}{FP+TN} \end{array} $$

4 $$ {{}{\begin{aligned} MCC = \frac{TP \times TN - FP \times FN}{\sqrt{(TP+FP) \times (TP+FN) \times (FP+TN) \times (TN+FN)}} \end{aligned}}}  $$

### In silico validation

After Granger Causality test and stepwise character selection, a predictor set that contained the least genes but performs well classification accuracy was extracted. In order to verify the effectiveness of the results, we downloaded 6 independent gene expression profiles (GSE10072, GSE83213, GSE2088, GSE32863, GSE43458, GSE27262) provided by GEO database (See Table 1).

Precision was measured by Eqs. 1, 2, 3, 4. Receiver Operating Characteristic (ROC) curves and Area Under Curve (AUC) facilitated the display of ultimate performance.

## Result

### Diff-genes detection

By applying DEA in gene, methylation, and miRNA microarrays, a total of 6155 DEGs, 266 Dmets, and 325 DEmiRNAs were selected as diff-genes. Of the DEmiRNAs, 315 genes with degrees greater than 2 were identified as DEmiRNA-target-genes. The top 5 DEmiRNA-target-genes ordered by indegree are shown in Table 3. Numbers in brackets represent the number of genes regulated by the corresponding DEmiRNAs.

**Table 3:** Top 5 target genes and their top 4 regulator miRNA

Target genes	Degree	DEmiRNA
VEGFA	12	hsa-mir-378a (120), hsa-mir-373 (62), hsa-mir-34a (21), hsa-mir-17 (20)
CCND1	10	hsa-mir-34a (21), hsa-mir-17 (70), hsa-mir-449a (14), hsa-mir-19a (12)
CDK6	10	hsa-mir-615 (121), hsa-mir-21 (70), hsa-mir-203a (21), hsa-mir-34a (21)
BCL2	9	hsa-mir-375 (419), hsa-mir-429 (26), hsa-mir-34a (21), hsa-mir-17 (20)
PTEN	7	hsa-mir-21 (48), hsa-mir-19a (12), hsa-mir-217 (11), hsa-mir-144 (8)

**Fig. 4:**
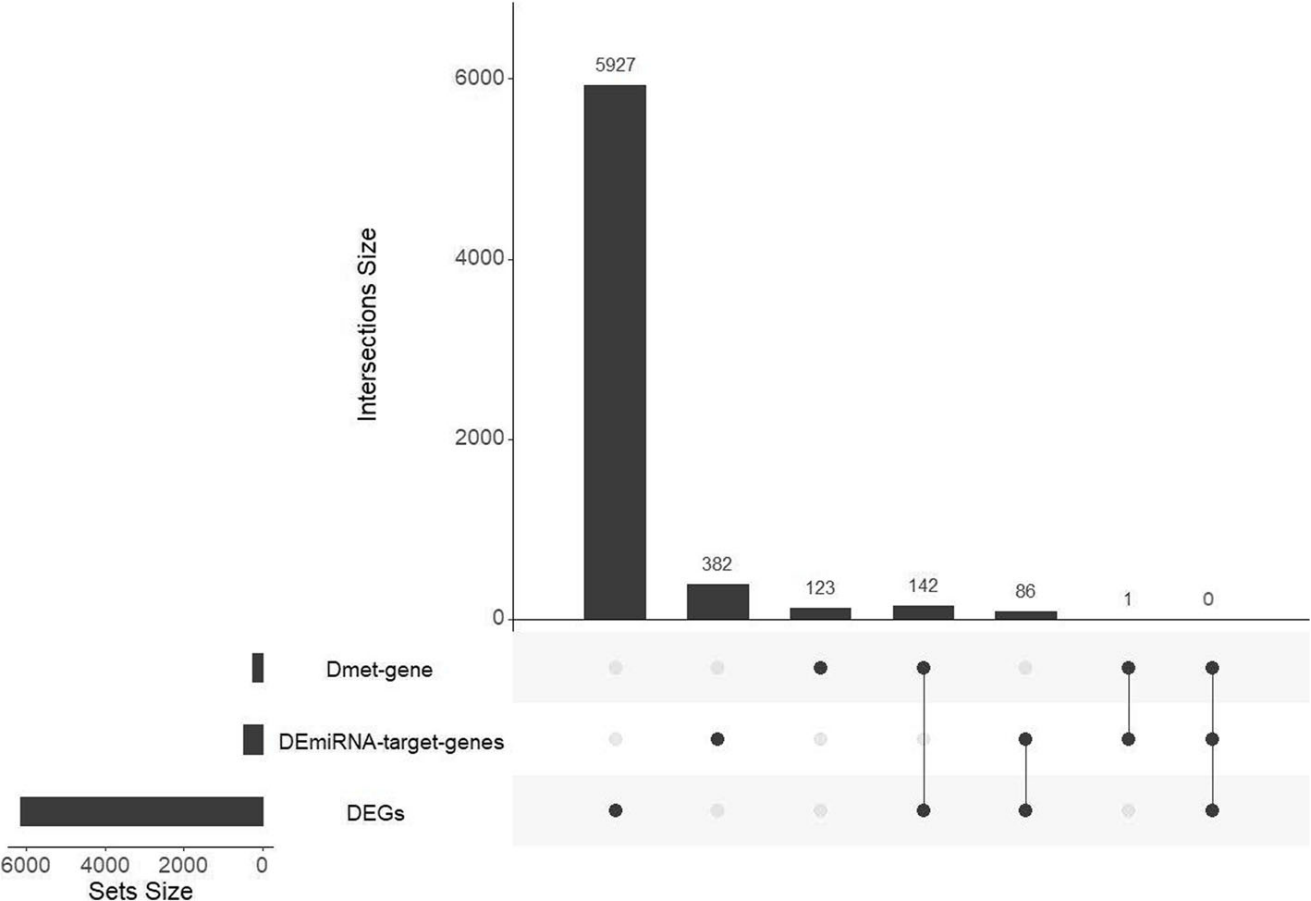
Distribution of the 6661 diff-genes. The lengths of horizontal bars represent the size of Dmet-located-gene, DEmiRNA-target-gene, and DEG. The heights of the vertical bars represent the size of intersections among Dmet-located-gene, DEmiRNA-target-gene, and DEG group

Distribution of the 6661 diff-genes is shown in Fig. 4. Most of the diff-genes were from the DEG group, and were not overlapping with those from Dmet-gene or DEmiRNA-target-gene group. 229 diff-genes were simultaneously from two groups. However, no genes were simultaneously from all the three groups.

### Feature genes screening

**Fig. 5:**
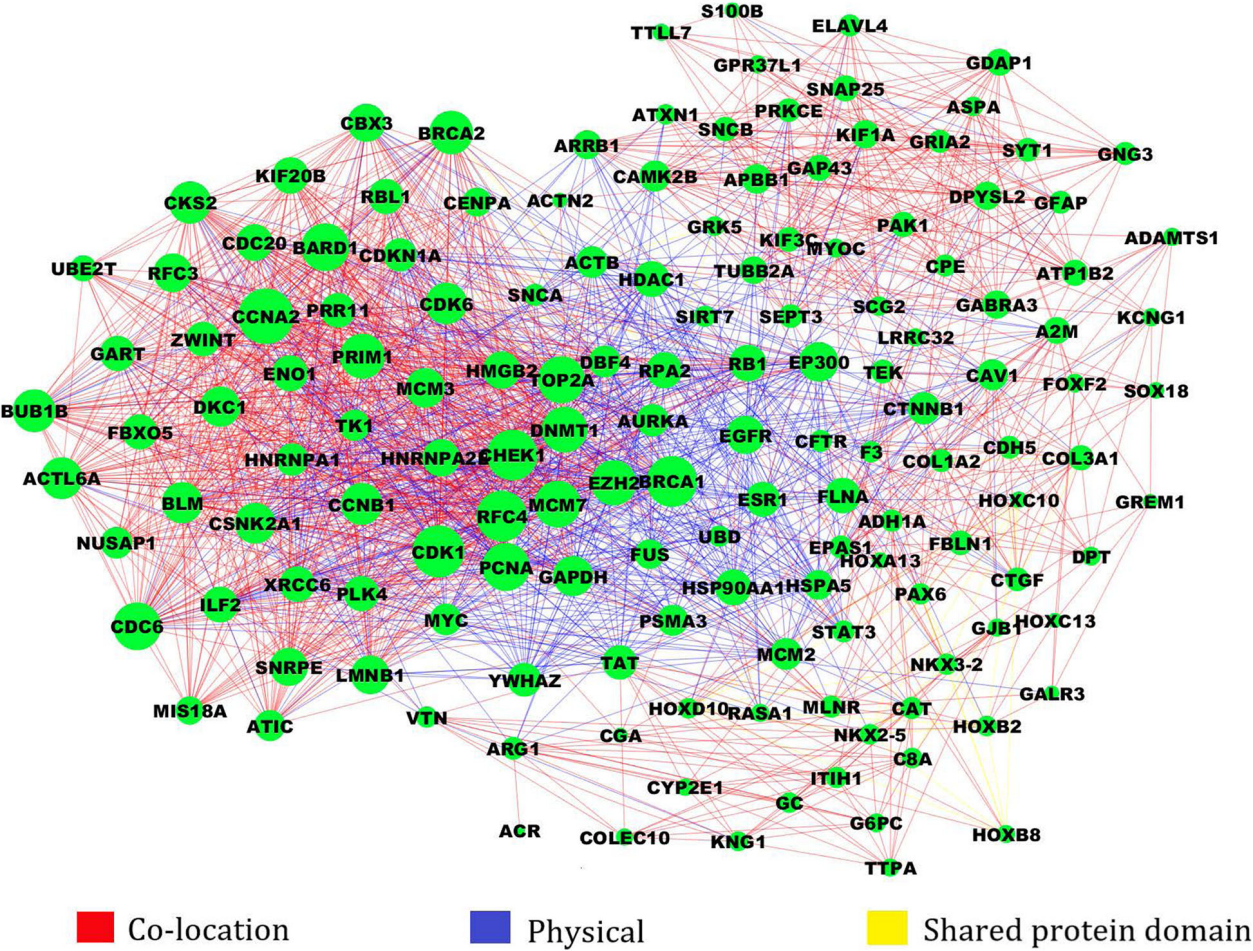
Multi-interaction network of 148 feature genes. The red lines indicate the co-location interactions, the blue lines indicate the physical interactions, the yellow lines indicate the shared protein domain interactions, and the size of node indicates the degree

Considering that nodes with higher degree in the network play more crucial roles than the others, we constructed a multi-interaction network integrating gene-gene interaction information. Totally 148 genes whose degree greater than 120 were selected as the feature genes. The interaction network of feature genes is shown in Fig. 5.

**Fig. 6:**
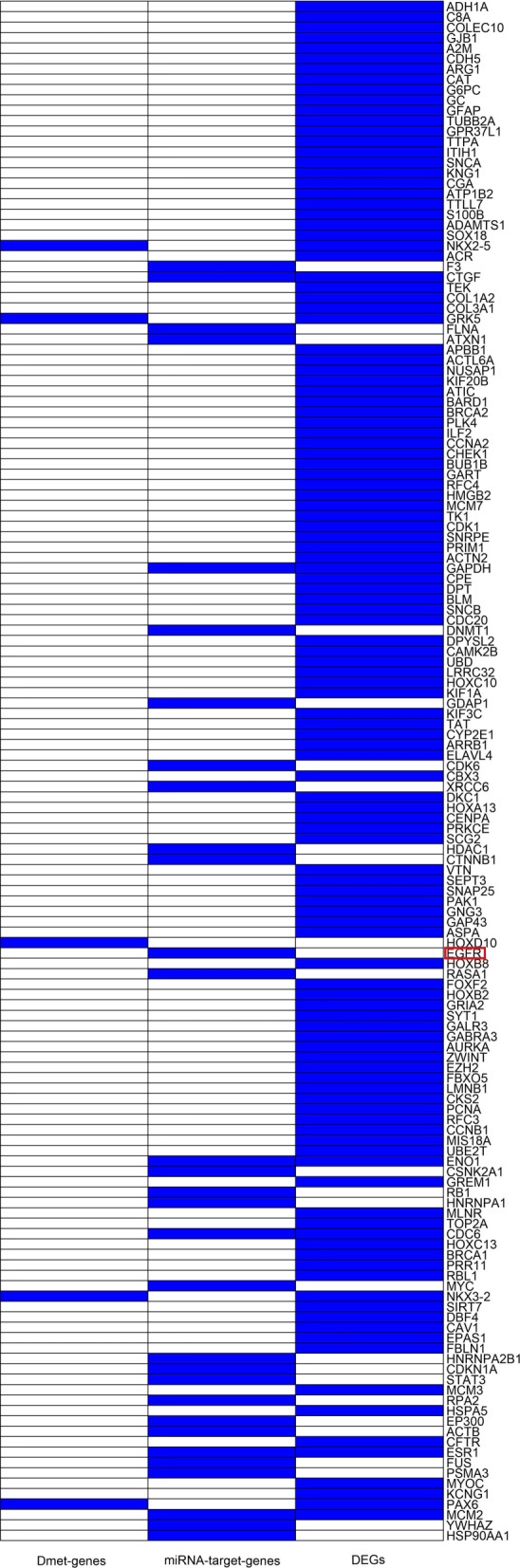
Sources of 148 feature genes. The blocks in blue denote the feature genes are DEGs, DEmiRNA-target-genes, or Dmet-genes. The gene highlighted by red square is *EGFR*, which is a well-known gene related to lung adenocarcinoma and it is also one of the predictors we identified. *EGFR* is only from DEmiRNA-target-gene group. This indicates that it is not sufficient to analysis gene activities based on single dataset

We screened the diff-genes using various expression microarrays instead of a single microarray. This is because any individual microarray is not sufficient. As revealed in Fig. 6, although most feature genes are DEGs, some important genes such as the well-known *EGFR* gene [37] are only included in DEmiRNA-target-gene group. This indicates the necessity of deriving predictors via analyzing gene’s performance based on various expression datasets.

### Gene predictors extraction

#### Granger causality test

**Fig. 7:**
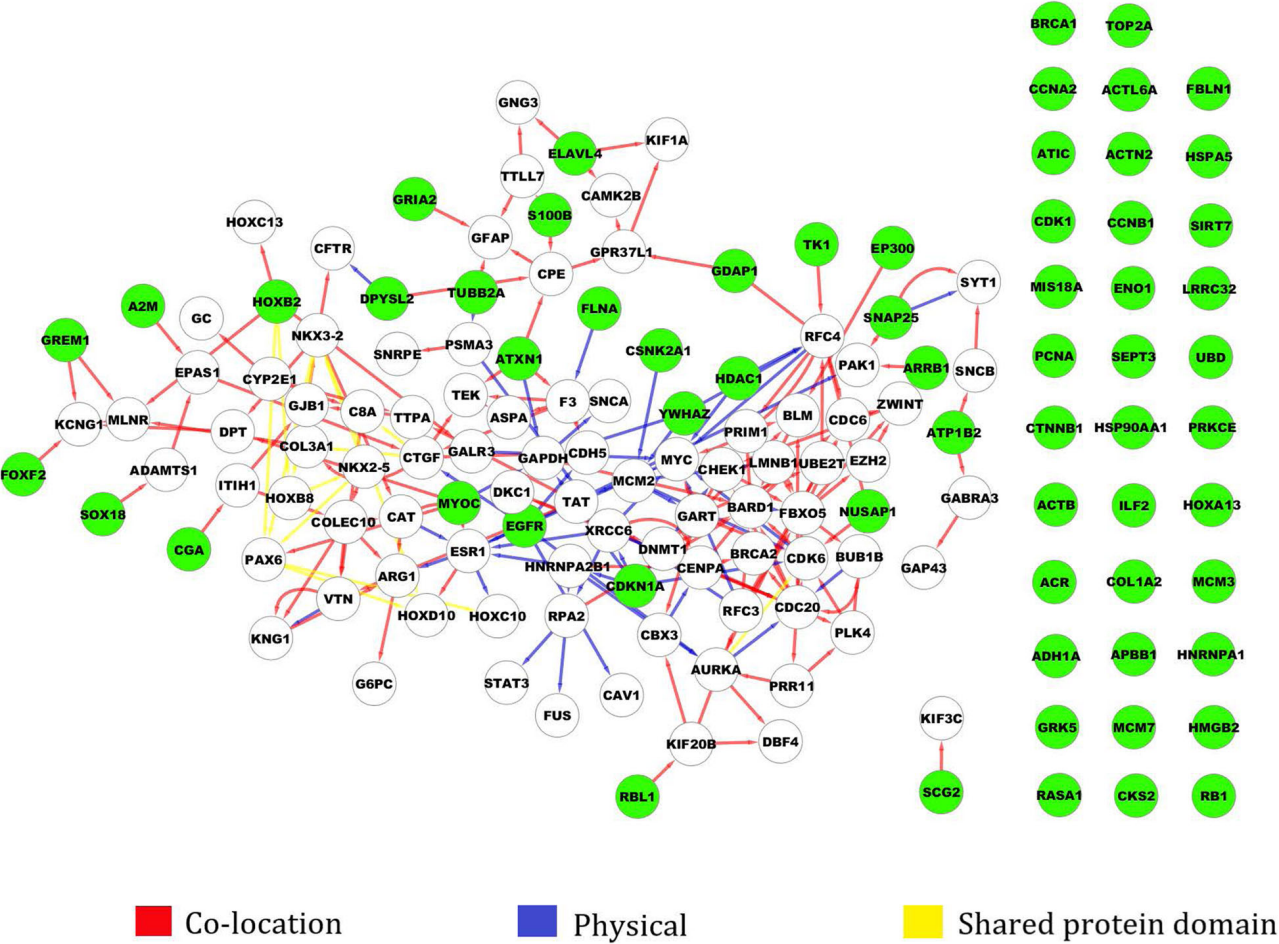
Directed causality network of 63 independent genes derived via Granger causality test. The directed arrows present regulation directions. The red lines indicate co-location interactions. The blue lines indicate physical interactions. The yellow lines indicate shared protein domain interactions. The nodes in green present the 63 globally independent genes screened

**Fig. 8:**
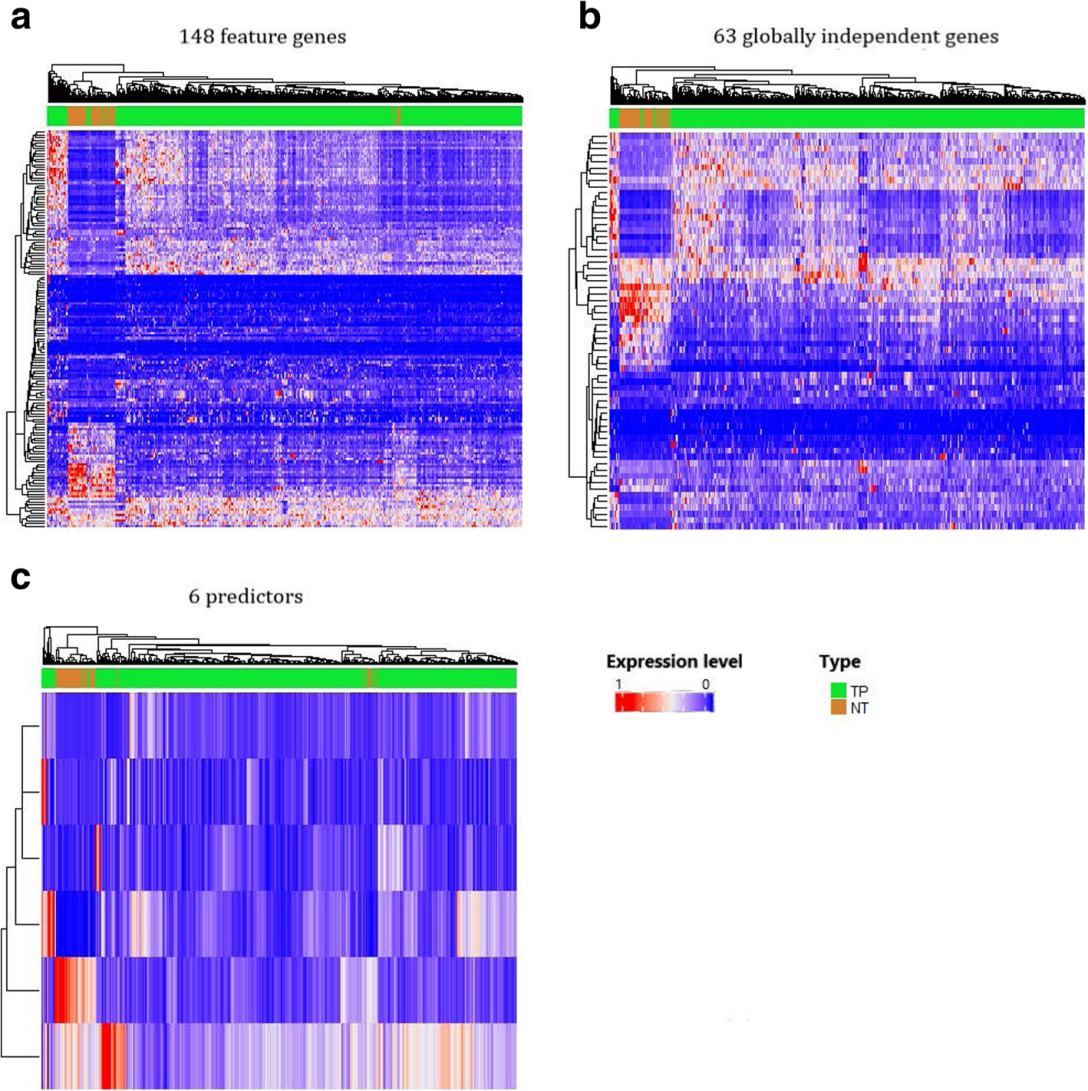
Performance of RF as a classifier based on 148 diff-genes, 63 feature genes, 6 predictors. **a**: 148 diff-genes, **b**: 63 feature genes, **c**: 6 predictors. “TP” and “NT” denote lung adenocarcinoma (marked in green) and normal samples (marked in brown), separately

In order to identify predictors from the feature genes based on the regulation relationship, we performed the Granger causality test (for flow chart see Fig. 1), and transformed the undirected interaction network to the directed causality network. Using the method for screening the globally independent genes, a total of 63 globally independent genes were selected. The causality network of those globally independent genes is shown in Fig. 7. The heatmaps (See Fig. 8b) show that the performance in classification achieved based on the independent genes (ACC: 98.7%) is better than that based on the 148 feature genes (See Fig. 8a, ACC: 96.6%).

During granger causality test, some interaction edges without granger causality relationship were removed. Of note, it does not mean that those edges are useless; on the contrary, they are important in biological processes. Most interactions are not of causality relationship according to our algorithm.

We further counted the number of edges that were tested as causality interactions for the three kinds of gene-gene interactions (i.e., co-location interaction, physical interaction, shared protein domain interaction). Such statistic was applied to three networks, namely, the globally independent gene interaction network, causality network and feature gene interaction network. The results are shown in Table 4. Percentages in the last column present the ratio of the edge quantity for one type of interaction in causality network to that in feature genes interaction network. All the percentages are lower than 50%, meaning that only a small part of interactions were tested as ganger causal interaction. Moreover, genes with shared protein domain interaction (40.6%) were more likely to be tested as of causality relation, compared with the other two kinds of interactions (11.6% for co-location interaction and 8.9% for physical interaction).

**Table 4:** Numbers of causality edges for each interaction type in three networks

Interaction type	Number of edges for each interaction type		
	Independent genes network	Causality network	Feature genes network
Co-location	37	142	1219 (11.6%)
Physical	2	52	581 (8.9%)
Shared protein domain	13	23	32 (40.6%)

Except the directed causality interaction, there are also some indirect relationship in the causality network. For example, gene *G*_1_ regulates *G*_2_ and *G*_2_ regulates *G*_3_, while *G*_1_ doesn’t directly regulate *G*_3_. To evaluate how such indirect causal relationship affects our results, we performed an experiment as follows. Let *G* denotes the resultant 63 globally independent genes, *D* denotes the directly dependent genes of the 63 independent genes (i.e., *G*), *I* denotes both the directly dependent genes of *D* and the indirect dependent genes of *G*. We then measured the classification performance of *G*, *D*, *I*, *G*∪*D* and *G*∪*I*. Their accuracies and AUCs are shown in Table 5, where the bold number represents the maximum value of the column.

**Table 5:** Classification performances of the 5 gene sets including *G*, *D*, *I*, *G*∪*D* and *G*∪*I*

Gene set	Number	Precision				AUC
		ACC (%)	SN (%)	SP (%)	MCC (%)	
*G*	63	98.7	88.7	96.6	93.5	0.89
*D*	42	97.4	96.5	91.8	88.1	0.88
*I*	26	96.6	95.7	90.1	85.5	0.91
*G*∪*D*	105	97.2	95.9	92.3	87.7	0.86
*G*∪*I*	89	97.6	96.3	94.3	90.0	0.89

From the table, both the ACC of *G*∪*I* and the ACC of *G*∪*D* are less than ACC of *G*. That is, *I* and *D* contain redundant information and ∖or interference information relative to *G*. Furthermore, the largest ACC is achieved by *G*. Considering the definition of granger causality, we believed that *G* could define both their directed independent genes and indirect independent genes. Thus, we only reserved *G* in this step.

#### Stepwise predictor selection based on Random Forest

The 63 globally independent genes are still too many in clinical diagnose. To further reduce the number and screen predictors, we performed the stepwise predictor selection method based on Random Forest classification model (See Fig. 3). A total of 6 predictors were uncovered in the end, namely, *TOP2A, GRK5, SIRT7, MCM7, EGFR, COL1A2*, with ACC up to 97.6%.

We performed the RF with 5-fold cross validation as classifier to measure the classification performance of the 6 predictors, the 63 globally independent genes, and the 148 feature genes separately. Considering the randomness of RF, the process was repeated 2000 times and calculated the average accuracy. The results are shown in Table 6.

**Table 6:** Classification performances of the 3 gene sets including feature genes, globally independent genes and predictors

Gene set	Number	Precision				AUC
		ACC (%)	SN (%)	SP (%)	MCC (%)	
Feature gene	148	97.4	95.1	95.0	87.5	0.90
Independent gene	63	97.7	88.7	96.6	93.5	0.89
Predictor	6	97.6	98.2	94.7	90.8	0.83

**Fig. 9:**
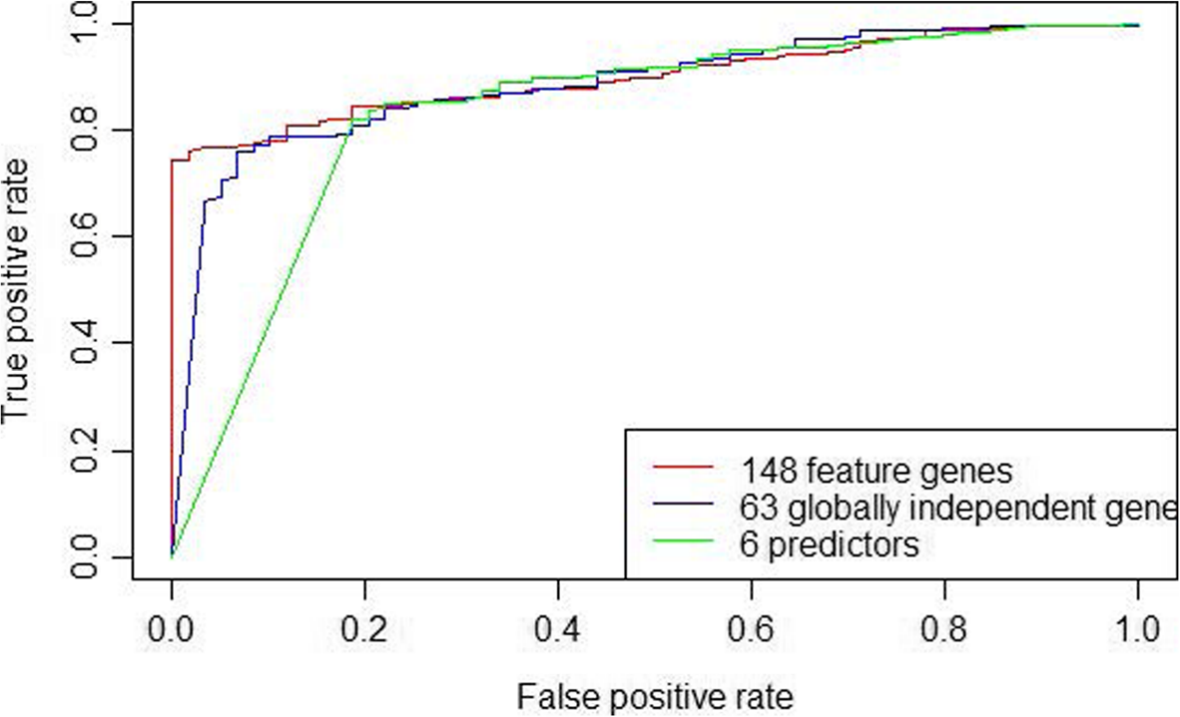
ROC curves of three gene sets

Compared with the 148 feature genes of which the ACC is 97.4%, the number of predictors is only 6 with the ACC up to 97.6%. Meanwhile, the resultant 6 predictors perform similar accuracy with the 63 globally independent genes (See Table 6). These results indicate that our method is efficient in character selection. Heatmaps (See Fig. 8a) and ROC curves (See Fig. 9) also show the well performance of the 6 predictors.

### In silico validation

**Fig. 10:**
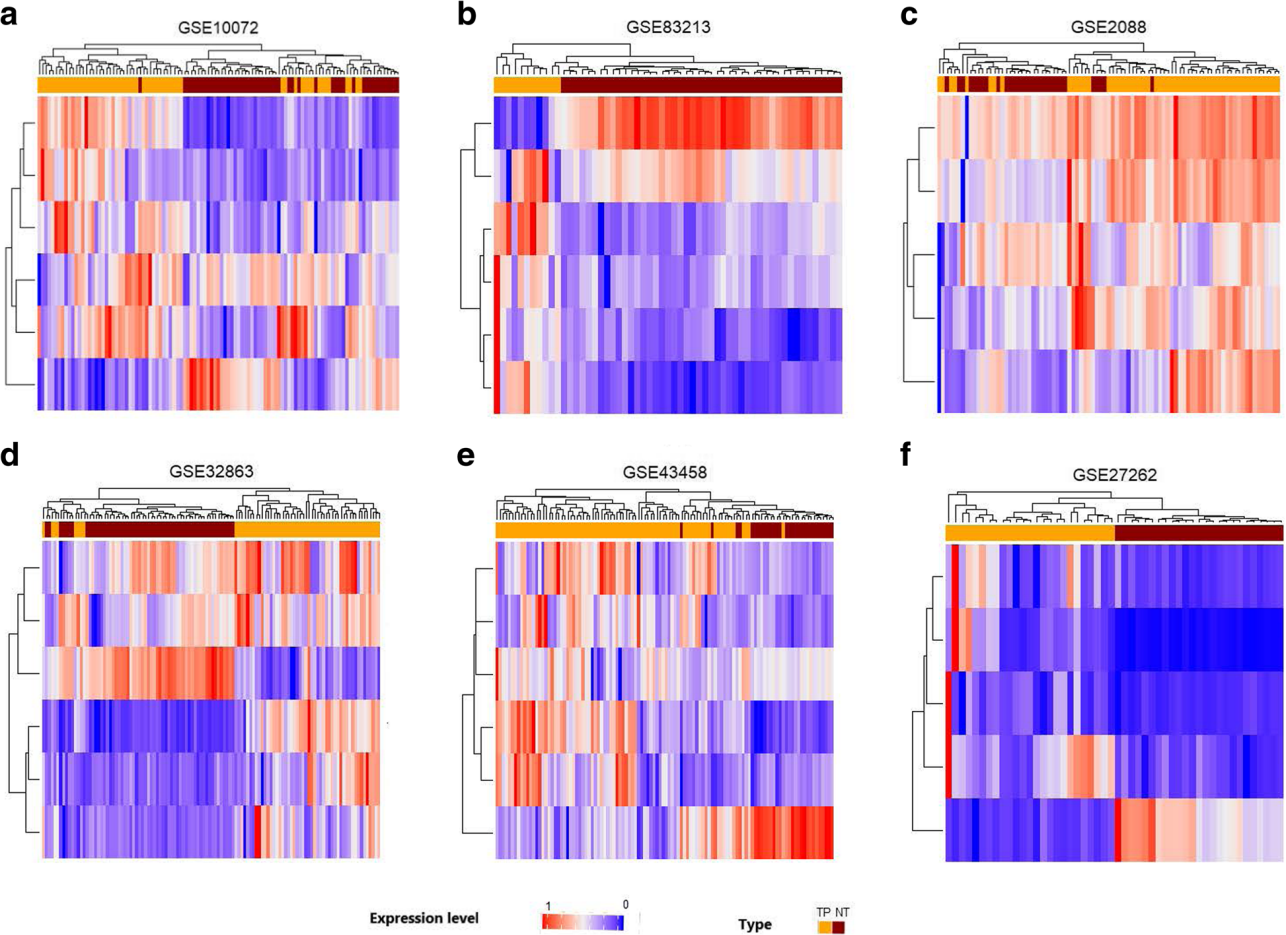
Validation in 6 datasets from different sources. **a**: GSE10072, **b**: GSE83213, **c**: GSE2088, **d**: GSE32863, **e**: GSE43458, **f**: GSE27262. “TP” and “NT” denote lung adenocarcinoma (marked in yellow) and normal samples (marked in darkred), respectively

We applied the 6 predictors in six independent validation datasets in GEO database, namely, GSE10072, GSE83213, GSE2088, GSE32863, GSE43458, GSE27262 (for more details see Table 1). The classification accuracies are shown in Table 7. The minimum ACC is achieved in GSE43458 (ACC: 95.3%) while the maximum is in GSE27262 (100%). Heatmaps of six validation datasets are shown in Fig. 10.

**Table 7:** Classification accuracies of the resulting 6 predictors in 6 datasets

Dataset	Tumor (%)	Precision			
		ACC (%)	SN (%)	SP (%)	MCC (%)
GSE10072	50 (47)	98.3	98.2	94.7	90.8
GSE83213	11 (19)	95.7	100	92.5	86.0
GSE2088	57 (66)	97.2	96.9	96.5	94.3
GSE32863	58 (50)	95.3	95.7	90.0	89.0
GSE43458	80 (110)	98.3	98.0	95.0	95.0
GSE27262	25 (50)	100	100	100	100

## Discussion

Although the occurrence and development of lung adenocarcinoma are complex, fast diagnose can be realized via analyzing the expression of predictor genes. In clinical diagnose, a proper predictor set should meet two criteria. One is higher prediction precision, and the other is fewer predictor members. In this paper, we proposed a two-step approach for extracting predictors based on expression microarrays, aiming to differentiate lung adenocarcinoma cancer samples vs normal samples.

Firstly, we exacted feature genes based on expression datasets. Considering that individual expression profiles are not enough for uncovering gene activities dynamically and systematically, we applied DEA to three microarrays (including gene, methylation and miRNA microarrays) for screening diff-genes. 148 feature genes were then selected from these diff-genes via conducting and analyzing the multi-interaction network of diff-genes. The predictors were then exacted by a two-step method. 63 globally independent genes that play dominant roles in the whole network were firstly screened, with the use of Granger causality test based on the undirected feature gene interaction network. To further reduce the number, we proposed a stepwise character extraction method based on Random Forest classification model. Finally, only 6 genes were identified as predictors, which are *TOP2A, GRK5, SIRT7, MCM7, EGFR, COL1A2*. The classification accuracy of these predictors is up to 98.3%.

To verify the performance of the 6 predictors in classifying cancer and normal samples, six datasets from different sources were applied. The accuracies were uncovered to be in the range from 95.7 to 100% (GSE10072: 98.3%, GSE83213 95.7%, GSE2088: 97.2%, GSE32863: 95.3%, GSE43458: 98.3%, GSE27262: 100%, Table 7). This approves the robustness of our approaches.

Of the 6 predictors, 5 genes including *TOP2A, SIRT7, MCM7, EGFR, COL1A2* are downregulated and 1 gene *GRK5* is upregulated, compared with normal samples. It should be mentioned that, *EGFR* is from the DEmiRNA-target-gene group only, *TOP2A, SIRT7, MCM7, and COL1A2* are from the DEG group only, and GRK5 is from both the DEG and Dmet-gene group. This suggests the necessity of screening diff-gens from multiple datasets. Additionally, all the predictors are associated with lung adenocarcinoma or cancer. *TOP2A* is an important gene that controls and alters the topologic states of DNA during transcription, and regulates cell cycle and p53 signaling pathways in some cancers [38]. Derita et al. [39] demonstrated that *GRK5* regulates the Src and IGF-IR signaling and have been implicated in cancer. Shi et al. [40] found *SIRT7* functions as an oncogene in non-small cell lung cancer. *EGFR* is a well-known gene that associated with lung adenocarcinoma [41, 42]. Misawa et al. [43] suggested the methylation of *COL1A2* is related to some cancers. Moreover, our method can be applied to other diseases for screening the predictors.
